# Danggui Shaoyao San ameliorates neuroinflammation in a D-galactose-induced Alzheimer’s disease rat model by suppressing the JAK2/STAT3 pathway and modulating Th17/Treg -related immune dysregulation

**DOI:** 10.3389/fcell.2026.1763180

**Published:** 2026-02-25

**Authors:** Gangying Fu, Shuyao Tang, Xin Sun, Jiajun Tong, Mengfen Zhou, Ping Li, Pan Meng, Shaowu Cheng, Zhenyan Song

**Affiliations:** 1 Key Laboratory of Hunan Province for Integrated Traditional Chinese and Western Medicine on Prevention and Treatment of Cardio-Cerebral Diseases, School of Integrated Chinese and Western Medicine, Hunan University of Chinese Medicine, Changsha, China; 2 National Key Laboratory Cultivation Base of Chinese Medicinal Powder & Innovative Medicinal Jointly Established by Province and Ministry, Changsha, China; 3 Academy of Chinese Medical Sciences, Hunan University of Chinese Medicine, Changsha, China

**Keywords:** Alzheimer’s disease, Danggui Shaoyao San, JAK2/STAT3 pathway, neuroinflammation, Th17/Treg

## Abstract

**Objective:**

This study investigates the therapeutic potential of Danggui Shaoyao San (DSS), a traditional Chinese herbal formula, focusing on its effects on Th17/Treg -associated immune regulation and the JAK2/STAT3 signaling pathway.

**Methods:**

Forty male Sprague-Dawley rats were randomly divided into five groups: control, AD model, low-dose DSS (12 g/kg/day, raw herbal materials), high-dose DSS (24 g/kg/day, raw herbal materials), and donepezil (0.5 mg/kg/day). AD models were established by intraperitoneal injection of D-galactose (100 mg/kg/day) for 8 consecutive weeks. Behavioral tests, flow cytometry, biochemical assays, histological analyses, qPCR, and Western blotting were used to evaluate DSS’s effects. Untargeted metabolomics profiled metabolic alterations, while network pharmacology and molecular docking were integrated to predict key targets and pathways.

**Results:**

DSS treatment significantly alleviated neuronal damage, suppressed neuroinflammation, and improved learning and memory deficits in AD rats. Moreover, DSS was associated with alterations in Th17- and Treg-related immune dysregulation both in the brain and periphery. Serum metabolomic identified disruptions lipid metabolism and amino acid metabolism pathways. Network pharmacology and experimental validation indicated that DSS exerts its anti-neuroinflammatory effects by inhibiting JAK2 and STAT3 phosphorylation, reducing their nuclear translocation, and consequently suppressing Th17 differentiation and pro-inflammatory cytokine production.

**Conclusion:**

DSS is a promising candidate for AD treatment, with neuroprotective and cognitive-enhancing properties mediated through immunomodulation and JAK2/STAT3 pathway inhibition.

## 1 Introduction

Alzheimer’s disease (AD) is a neurodegenerative disorder clinically characterized by memory impairment and cognitive dysfunction. Accounting for 60%–70% of all dementia cases, AD represents a significant global health challenge (Jia et al., 2018). The pathogenesis of AD remains incompletely understood but is widely accepted to involve a complex interplay of multiple pathological processes, including β-amyloid (Aβ) deposition, Tau protein hyperphosphorylation, neuroinflammation, and neuronal apoptosis (Leng and Edison, 2021). Although the U.S. Food and Drug Administration (FDA) has approved several drugs for AD treatment—such as cholinesterase inhibitors, memantine, and novel anti-Aβ antibodies—these therapies only alleviate symptoms without effectively halting disease progression. Additionally, their use is often limited by adverse effects including dizziness, hallucinations, and hepatotoxicity, resulting in suboptimal long-term efficacy (Alzheimer’s disease facts and figures, 2024).

In recent years, neuroinflammation has been recognized as a critical pathological driver of neurodegeneration in AD (Leng and Edison, 2021). In this context, Aβ plaques activate microglia, triggering the release of pro-inflammatory cytokines such as interleukin-1β (IL-1β), IL-6, and tumor necrosis factor-α (TNF-α). This inflammatory response exacerbates Aβ accumulation and Tau pathology, establishing a vicious cycle of chronic inflammation that ultimately leads to synaptic damage and neuronal death (Sun et al., 2023). Emerging evidence also implicates the peripheral immune system, particularly T lymphocytes, in AD-related neuroinflammation. Among these, the balance between T helper 17 (Th17) cells and regulatory T (Treg) cells is crucial. Th17 cells secrete pro-inflammatory cytokines, including IL-17A, which exacerbate inflammation, whereas Treg cells produce anti-inflammatory cytokines such as IL-10 and transforming growth factor-β (TGF-β), maintaining immune homeostasis. Studies have identified a disrupted Th17/Treg ratio in the brains of AD patients and animal models, characterized by increased Th17 cells and related inflammatory mediators alongside decreased Treg cells and anti-inflammatory factors (Zhang et al., 2021). However, the precise contribution of Th17/Treg-associated immune dysregulation to neuroinflammation in AD remains to be fully elucidated.

The Janus kinase/Signal Transducer and Activator of Transcription (JAK/STAT) signaling pathway plays a central role in regulating T cell differentiation and function, particularly in maintaining the Th17/Treg equilibrium (Xin et al., 2020). In AD, aberrant activation of the JAK2/STAT3 pathway promotes pro-inflammatory microglial polarization and cytokine release. It also disrupts immune homeostasis by upregulating RORγt, the key transcription factor for Th17 cells, and downregulating Foxp3, the transcription factor essential for Treg cells, thereby contributing to cognitive decline (Chiba et al., 2009).

Given the multifactorial nature of AD, Traditional Chinese Medicine (TCM) offers unique advantages through its multi-component and multi-target holistic approach (Kirubakaran, 2025). DSS, a classic formula documented in *Synopsis of the Golden Chamber*, has been widely used in TCM practice for dementia and memory impairment (Yunhui, 2020). Modern pharmacological studies demonstrate that DSS significantly improves cognitive function in various AD animal models and attenuates Aβ-induced synaptic damage and neuronal apoptosis (Fu et al., 2016). The neuroprotective effects of DSS are closely linked to its anti-inflammatory properties. Preliminary evidence indicates that DSS may reduce neuroinflammation by inhibiting signaling pathways such as NLRP3/Caspase-1 and NF-κB (Zhenyan, 2021). However, whether DSS modulates peripheral and central Th17/Treg immune balance and its relationship with the JAK2/STAT3 pathway remains unclear. The D-galactose-induced aging-related cognitive impairment and senile neuroinflammation rat model replicates core features of AD, including increased brain inflammation, oxidative stress, and cognitive deficits (Gao et al., 2015; Rehman et al., 2017).

In this study, we employed this model to evaluate the neuroprotective effects of DSS against AD-associated neuroinflammation. Moreover, by integrating network pharmacology, serum metabolomics, and molecular biology techniques, we aimed to elucidate the potential mechanisms of DSS, focusing on the regulation of Th17/Treg -related immune responses and the JAK2/STAT3 signaling pathway.

## 2 Materials and methods

### 2.1 Animals

Forty male Sprague-Dawley rats (6–8 weeks old, SPF grade) were purchased from Hunan Slake Jinda Experimental Animals Co., Ltd. (SCXK (Xiang) 2021-0002). Rats were housed in the Animal Experiment Center of Hunan University of Chinese Medicine under controlled conditions (25 °C ± 2 °C, 12 h light/dark cycle), with free access to standard chow and water. All experimental procedures were approved by the Ethics Committee of Hunan University of Chinese Medicine. (ethical approval code: HNUCM21-2312-24).

### 2.2 Compounds and reagents

Medicinal materials for Danggui Shaoyao San (DSS) including *A. sinensis (Oliv.) Diels* (Danggui) (#CK25111301), *Paeonia lactiflora Pall* (Shaoyao) (#HH25111401), *Atractylodes macrocephala Koidz* (Baizhu) (#CK25110302), *Poria cocos (Schw.) Wolf* (Fuling) (#HH25111101), *Alisma orientalis (Sam.) Juzep* (Zexie) (#HH25110505), and *Ligusticum chuanxiong Hort* (Chuanxiong) (#HH25102701) were obtained from the Traditional Chinese Medicine Pharmacy of the First Affiliated Hospital of Hunan University of Chinese Medicine. Other reagents included sodium pentobarbital (Merck KGaA, Germany, #P3761), donepezil hydrochloride (MCE, United States, #HY-B0034), D-galactose (Sigma-Aldrich, Shanghai, China, #V900922), primary antibodies targeting STAT3 and Foxp3 (Proteintech, Wuhan, China, #102532-AP, #22228-1-AP), ROR-γt and p-STAT3-Y705 antibodies (HUABIO, Hangzhou, China, #HA722121, #ET1603-40), JAK2 antibody (Aifang, Hunan, China, #AFRM99330), p-JAK2-Y1007/1008 antibody (ABclonal, Wuhan, China, #AP0531), β-actin antibody (Affinity Biosciences, China, #AF7018), postsynaptic density protein 95 (PSD95) and synaptophysin I antibody (abcam, Cambridge, United Kingdom, #ab18258,#ab254349), secondary antibodies (Elabscience, Wuhan, China, #E-AB-1003), Iba1 antibody (Wako Pure Chemical Industries, Ltd., Japan, #011-27991), primers for β-actin, IL-1β, IL-6, etc., were designed using Primer 6.0 software based on sequences from the NCBI database and synthesized by Sangon Biotech (Shanghai), China, reverse transcription kit (Novoprotein, Suzhou, China, #E047-01B), SYBR Mixture premix and RIPA protein lysis buffer (Comwin Biotech, Jiangsu, China, #CW3008M, #CW2333S), ELISA kits (Jianglai Biotechnology, Shanghai, China, #JL13427, #JL20880), BCA protein concentration assay kit (Elabscience Biotechnology, Wuhan, China, #E-BC-K318-M), TRIzol, SlowFade™ Gold Antifade Mountant with DAPI, and donkey anti-goat Alexa Fluor 647 fluorescent secondary antibody (red) (Thermo Fisher Scientific, United States, #15596-026, #S36942, #A32849), universal two-step detection kit (Zhongshan Golden Bridge, Beijing, China, #PV-9000), Toluidine blue, Triton X-100, and antifade mounting medium with DAPI (Solarbio Science & Technology Co., Ltd., Beijing, China, #G3663, #T8200, #S2110), hematoxylin staining solution and eosin staining solution (Zhongshan Golden Bridge Biotechnology Co., Ltd., Beijing, China, #ZLI-9610, #ZLI-9613), Fixation/permeabilization kits, dyes, and antibodies against CD4, Foxp3, and IL-17A for phenotypic analysis of Th17 and Treg cells were purchased from BD Pharmingen (United States), detailed information is provided in Supplementary Table S1.

### 2.3 Preparation of DSS

The composition of DSS was based on the original ratios from the *Synopsis of Prescriptions of the Golden Chamber* by Zhang Zhongjing (Danggui 9 g, Baishao 48 g, Baizhu 12 g, Fuling 12 g, Zexie 24 g, and Chuanxiong 24 g), referring to the previous study (Song et al., 2021). The herbal mixture was extracted by decoction with water and concentrated using a rotary evaporator (Hei-VAP ML, Heidolph, China) to yield a final concentration of 5.16 g/mL. Quality control information for the DSS was reported by our group using liquid chromatography-tandem mass spectrometry (LC-MS/MS) (Song et al., 2020).

### 2.4 Animal grouping and treatment

**FIGURE 1:**
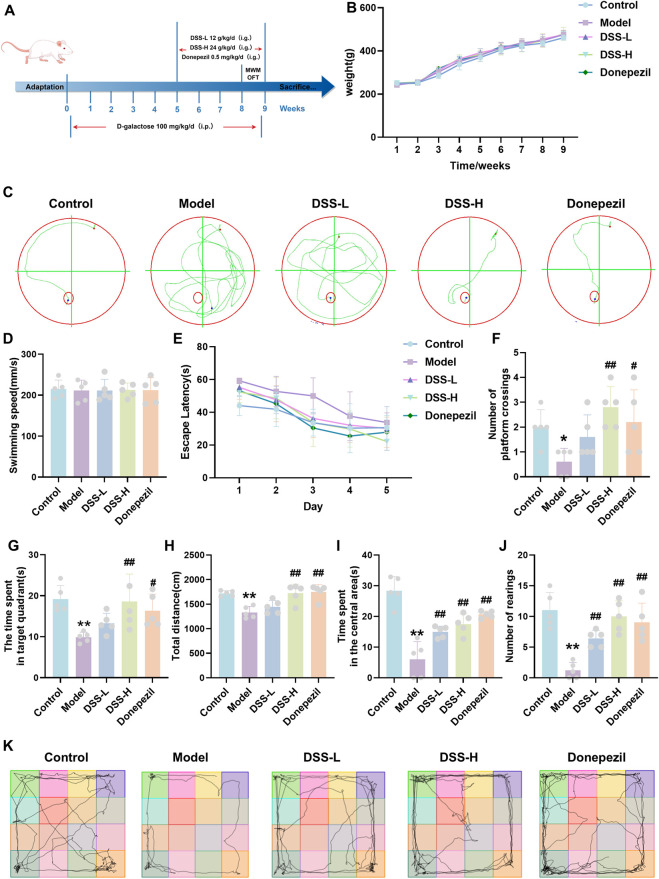
DSS ameliorates learning and memory impairments in D-galactose-induced AD rats. **(A)** Flowchart of the animal experiment. **(B)** Body weight changes over 9 weeks. **(C)** Representative movement trajectories of rats from each group during the navigation phase of the MWM test. **(D)** Swimming speed in the spatial probe trial. **(E)** Changes in escape latency during the training period for each group. **(F)** Number of crossings over the original platform location in the spatial probe trial. **(G)** Time spent in the target quadrant by rats. **(H)** Total movement distance of rats in the open field. **(I)** Time spent in the central area. **(J)** Number of entries into the central area. **(K)** Representative movement trajectories of rats from each group in the OFT. All data are presented as mean ± SD, n = 5. *p < 0.05, **p < 0.01 vs. control group; #*p* < 0.05, ##*p* < 0.01 vs. model group. MWM: Morris water maze; OFT: Open field test.

Forty male Sprague-Dawley rats were randomly assigned into five groups (n = 8 per group): control, D-gal model (100 mg/kg), low-dose DSS (L-DSS, 12 g/kg/day, 0.9 mL/day concentrated extract), high-dose DSS (H-DSS, 24 g/kg/day, 1.8 mL/day), and donepezil (0.5 mg/kg/day) (Ayoub et al., 2022) groups. The dosage regimen was determined based on clinical equivalents. The low dose was calculated using the formula: Rat dose = 6.25 × (Clinical dose/Average human body weight of 65 kg). The high-dose group received twice the volume/concentration of the low-dose group (YMe, 2023). Except for the control group, all rats received intraperitoneal injections of D-galactose for 8 weeks to induce an AD-like model (Rehman et al., 2017), which is widely used to induce aging-related oxidative stress and neuroinflammation accompanied by learning and memory deficits. Drug administration started at week 5 and continued for 4 weeks. After behavioral analysis, the rats were sacrificed for subsequent experiments. Control rats received equal volumes of saline via gavage (Figure 1A). During the treatment period, animals were closely monitored for general health status, including body weight, food intake, locomotor activity, and coat condition.

### 2.5 Open field test (OFT)

The open field apparatus measured 100 cm × 100 cm × 40 cm, with black-painted inner walls and the floor divided into 16 squares. The central area consisted of 4 squares surrounded by 12 peripheral squares. Each rat was placed gently in the center and allowed to explore freely for 5 min. Between trials, the apparatus was cleaned with alcohol to remove odors. Parameters recorded included total distance traveled, number of entries into the central zone, time spent in central and peripheral zones, and movement trajectory. Data were collected and analyzed using Tracking Master V3.0 (XR-XZR209, Xinruan, Shanghai, China) (Kraeuter et al., 2019).

### 2.6 Morris water maze (MWM) test

The water maze pool was divided into four quadrants with distinct visual cues. The hidden platform was submerged 1 cm below the water surface in the target (first) quadrant. Training was conducted over 5 days, with four trials per day from different entry points and a maximum swim time of 60 s. Escape latency (time to reach platform) was recorded. Rats failing to find the platform within 60 s were guided to it for 10 s to facilitate learning. On day 6, the platform was removed for the probe test. Rats were released from the quadrant opposite the original platform location. The number of platform crossings, time in the target quadrant, and swim paths and swimming speed were recorded and analyzed using SuperMaze software (XR-XM101, Xinruan, Shanghai, China) (Vorhees and Williams, 2006).

### 2.7 Sample collection

Following anesthesia with pentobarbital sodium via intraperitoneal injection (40 mg/kg (Reid et al., 2003; Soma, 1983), dissolved in 0.9% NaCl), rats were fixed supine, and a U-shaped abdominal incision was made. Approximately 3 mL blood was collected from the abdominal aorta. The left ventricle was perfused with precooled physiological saline (4 °C), and the right atrium was cut open. Successful perfusion was confirmed by the whitening of visceral organs. All rats were humanely euthanized by rapid decapitation in accordance with approved institutional ethical guidelines. Brains were rapidly extracted; the left hemisphere was fixed in 4% paraformaldehyde for histology, while the hippocampus from the right hemisphere was dissected, snap-frozen in liquid nitrogen, and stored at −80 °C for molecular assays. The spleen was excised, cleared of connective tissue and fat, and placed in cold tissue preservation solution (4 °C) for flow cytometry.

### 2.8 Network pharmacology

Active components of DSS were screened from the TCMSP (Ru et al., 2014) and PubChem (Kim et al., 2021) databases using the botanical names as keywords, with selection criteria of oral bioavailability ≥30% and drug-likeness ≥0.1. Potential targets of these components were predicted using SwissTargetPrediction. Disease-related targets associated with neuroinflammation were obtained from GeneCards (Stelzer et al., 2011) and OMIM (Amberger et al., 2019). Intersection targets were identified using Venny online tool and imported into STRING (Szklarczyk et al., 2019) for protein-protein interaction network construction. Core targets were screened by topological analysis in Cytoscape with plugins Network Analyzer and cytoNCA. Gene Ontology (GO) and KEGG pathway enrichment analyses were performed via DAVID (Liu and Thomas, 2019; Kanehisa et al., 2017). An active component–core target–pathway network was constructed in Cytoscape 3.7.2 (Kohl et al., 2011). Software details and URLs are listed in Supplementary Table S2.

### 2.9 Molecular docking

The top 10 key active components of DSS were selected for docking with four target proteins (JAK2, STAT3, IL-10, IL-17) based on KEGG pathway analysis. Crystal structures were retrieved from the RCSB PDB database and preprocessed via PyMOL by removing ligands and water molecules, followed by structural optimization. Ligand structures were optimized using Chem3D. Docking was performed using CB-Dock2 online platform to obtain binding affinity scores (Vina Scores) and optimal conformations. Binding affinity scores less than −5.0 kcal/mol indicated strong binding. Scores were visualized and statistically analyzed on MicroBioinformatics platform. PyMOL 3.0 was used to visualize interactions with high binding affinity (Burley et al., 2021; Khanal et al., 2022).

### 2.10 Metabolomics

Untargeted serum metabolomics was conducted following established protocols (He et al., 2023). Serum samples were thawed at 4 °C, and 100 µL aliquots were mixed with 400 µL pre-cooled methanol-acetonitrile (1:1, v/v) for protein precipitation. After vortexing, samples were incubated at −20 °C for 1 h, centrifuged at 14,000 × g for 20 min at 4 °C, and supernatants collected for LC-MS analysis. Chromatographic separation used a Waters ACQUITY UPLC HSS T3 column (2.1 × 100 mm, 1.8 µm) at 40 °C with mobile phases A (water +0.1% formic acid) and B (acetonitrile +0.1% formic acid), flow rate 0.3 mL/min, injection volume 2 µL. Mass spectrometry was performed on Thermo Q Exactive HF-X with full scans in positive and negative ion modes (m/z 70–1050). Data processing included peak extraction, alignment, and normalization via Progenesis QI. PCA and OPLS-DA were employed for group discrimination. Differential metabolites met criteria of VIP >1.0, p < 0.05 (t-test), and fold change >1.5 or <0.67. Metabolite annotation and pathway enrichment were conducted using HMDB and KEGG databases.

### 2.11 Histological staining

After fixation and paraffin embedding, for each hemisphere, a series of 30 consecutive coronal sections (5 µm thick) encompassing the entire dorsal hippocampus were collected for analysis. HE staining: sections were baked, dewaxed, stained with hematoxylin (5 min), differentiated in hydrochloric acid-ethanol, blued in distilled water, dehydrated through graded alcohols, cleared in xylene, and mounted with neutral balsam. Nissl staining: sections were dewaxed, stained with 1% toluidine blue at 37 °C for 25 min, differentiated with 95% ethanol (30 s), dehydrated, and mounted. Images were captured using a Motic BA410E microscope (Motic, China) to evaluate hippocampal histology and neuronal Nissl body distribution.

### 2.12 RT-qPCR analysis

Total RNA was extracted from right hippocampal tissue using TRIzol reagent. RNA concentration and purity were measured before reverse transcription to cDNA using a commercial kit. qPCR was performed on a Bio-Rad CFX96 system with SYBR Green dye under the following conditions: initial denaturation at 95 °C for 5 min, followed by 40 cycles of 95 °C for 30 s and 58 °C for 30 s. Reactions were run in triplicate. β-actin served as the internal reference gene, and relative expression levels were calculated by the 2^−ΔΔCt^ method. Primer sequences are listed in Table 1.

**TABLE 1:** Primer sequences used in RT-qPCR analysis.

Gene	Forward sequence (5′-3′)	Reverse sequence (5′-3′)	Length (bp)
IL-10	GGT​TGC​CAA​GCC​TTG​TCA​G	CAT​TCT​TCA​CCT​GCT​CCA​CTG	199
IL-17A	TGA​AGT​GGA​ACG​GTT​GAG​GTA	GAT​GCT​GTT​GCT​GCT​ACT​GAA	193
IL-4	CAA​GGA​ACA​CCA​CGG​AGA​AC	GAC​CGC​TGA​CAC​CTC​TAC​A	147
RORγt	TAC​GCC​TGG​AGG​ACC​TTC​TA	ACA​TTC​TGA​CGA​GGA​CGA​CTT	237
IL-1β	CTC​ATT​GTG​GCT​GTG​GAG​AAG	ACA​CTA​GCA​GGT​CGT​CAT​CAT	148
IL-6	CCA​GCC​AGT​TGC​CTT​CTT​G	AAT​TAA​GCC​TCC​GAC​TTG​TGA​A	139
β-Actin	TCA​GGT​CAT​CAC​TAT​CGG​CAA​T	ACT​GTG​TTG​GCA​TAG​AGG​TCT​T	159
TGF-β	CGC​AAC​AAC​GCA​ATC​TAT​GAC	ACC​AAG​GTA​ACG​CCA​GGA​AT	204
FOXP3	CAC​GCA​TGT​TCG​CCT​ACT​TC	CTC​ACT​CTC​CAC​TCG​CAC​AA	101

### 2.13 Western blot

Hippocampal tissues from rats were collected, and total protein was extracted using RIPA lysis buffer. Protein concentration was quantified by BCA assay. Equal amounts of protein (30 μg) were separated by SDS-PAGE and transferred onto membranes. Membranes were blocked with 5% skim milk (or 5% BSA for phosphorylated antibodies) at room temperature for 1 h, followed by overnight incubation at 4 °C with primary antibodies: PSD95 (1:2000), synaptophysin I (1:1500), RORγt (1:1000), Foxp3 (1:1000), p-STAT3 (1:5000), STAT3 (1:10,000), p-JAK2 (1:500), JAK2 (1:5000), and β-actin (1:5000). After washing, membranes were incubated with HRP-conjugated secondary antibodies (1:10,000) for 1 h at room temperature. Chemiluminescent signals were detected using a Bio-Rad ChemiDoc XRS + imaging system, and band intensities were quantified with ImageJ software.

### 2.14 Immunofluorescence

Paraffin-embedded dorsal hippocampal sections were dewaxed, hydrated, and subjected to antigen retrieval. Sections were permeabilized with 0.3% Triton X-100 and blocked with 5% BSA for 30 min at room temperature. Primary antibodies against Iba-1, IL-17A, FOXP3, and p-JAK2 (each 1:50) were applied overnight at 4 °C. After washing with PBS, appropriate fluorescent secondary antibodies (1:500) were incubated for 1 h at room temperature in the dark. Nuclei were counterstained with DAPI, and sections were mounted with antifade medium. Images were acquired using the TissueFAXS Plus system, and positive cells were quantified.

### 2.15 Immunohistochemistry

Brain sections (5 μm) were prepared using a paraffin microtome. After dewaxing, hydration, and antigen retrieval, endogenous peroxidase activity was quenched with 3% H_2_O_2_. Sections were blocked with 5% BSA and incubated overnight with anti-p-STAT3 antibody (1:500) at 4 °C. After washing, secondary antibody (1:500) was applied for 30 min at room temperature. DAB was used for color development, followed by hematoxylin counterstaining, dehydration, clearing, and mounting. Images were captured under a light microscope, and positive staining was quantified.

### 2.16 ELISA

Collected arterial blood was allowed to clot at room temperature for 2 h, then centrifuged at 3,000 rpm for 10 min. Serum levels of IL-17A and IL-10 were measured according to the manufacturer’s instructions using ELISA kits. Absorbance was read on a microplate reader, and cytokine concentrations were calculated based on standard curves.

### 2.17 Flow cytometry

Spleens were harvested aseptically, minced, and filtered to obtain single-cell suspensions. After red blood cell lysis, cells were washed and resuspended in PBS. Surface staining with anti-CD4 antibody was performed at 4 °C for 30 min in the dark. Following permeabilization, intracellular staining for Foxp3 and IL-17 was conducted for 1 h at 4 °C. Cells were washed, resuspended in staining buffer, and analyzed on a BD LSRFortessa flow cytometer to quantify CD4^+^Foxp3^+^ Treg and CD4^+^IL-17^+^ Th17 cell populations.

### 2.18 Data analysis

Statistical analyses were conducted using SPSS 27.0 and GraphPad Prism 9.5.0. Data are presented as mean ± standard deviation (SD). For *in vivo* experiments, the number of biological replicates (n) refers to the number of individual animals used in each group. Behavioral assessments were conducted using independent cohorts of animals (n = 5 per group), while biochemical, histological, flow cytometry, and molecular analyses were performed using tissue samples randomly collected from the five animals (n = 3 per group). For normally distributed data with homogeneity of variance, one-way ANOVA was used; otherwise, the Kruskal–Wallis H test was applied. MWM acquisition data were analyzed using two-way repeated-measures ANOVA, with group as the between-subject factor and training day as the within-subject factor. Statistical significance was set at *p* < 0.05.

## 3 Results

### 3.1 DSS improves learning and memory in AD rats

To investigate the effects of DSS on neuroinflammation and cognitive function in AD rat model, we established D-galactose-induced AD rats, with the experimental timeline depicted in Figure 1A (By Fig draw). During the treatment period, animals were closely monitored for general health status, including body weight, food intake, locomotor activity, and coat condition. No signs of overt toxicity or abnormal behavior were observed in DSS-treated groups. Body weight changes over time have now been included in the revised manuscript (Figure 1B). Our team has performed comprehensive preclinical toxicological assessments, which indicate that DSS is a safe pharmaceutical formulation (Luo et al., 2024; Wang et al., 2023). MWM testing revealed that, compared with controls, model rats exhibited disorganized swimming trajectories during the navigation phase, reflecting impaired spatial exploration (Figure 1C). No significant differences in swimming speed were observed among groups on day 6, indicating that motor ability and general motivation during the task were comparable (Figure 1D). The model group demonstrated prolonged escape latency throughout training, indicative of deficits in spatial learning and memory (Figure 1E). Conversely, rats treated with low- and high-dose DSS (DSS-L, DSS-H) as well as donepezil showed markedly reduced escape latencies. In the spatial probe trial, the model group manifested a significant decline in both platform crossings (*p* < 0.05) and time spent in the target quadrant (*p* < 0.01), effects that were significantly reversed by DSS-H treatment (*p* < 0.01; Figures 1F,G). Open Field Test (OFT) results demonstrated that the model group exhibited reduced total movement distance, fewer entries into the central area, and decreased time spent therein (*p* < 0.01), indicative of diminished spontaneous locomotion. The treatment groups (DSS-H, donepezil) significantly ameliorated these behavioral deficits compared to the model group (*p* < 0.01; Figures 1H–K), suggesting that DSS improves behavioral performance in AD rats.

### 3.2 DSS mitigates neuronal damage and neuroinflammation in AD rats

**FIGURE 2:**
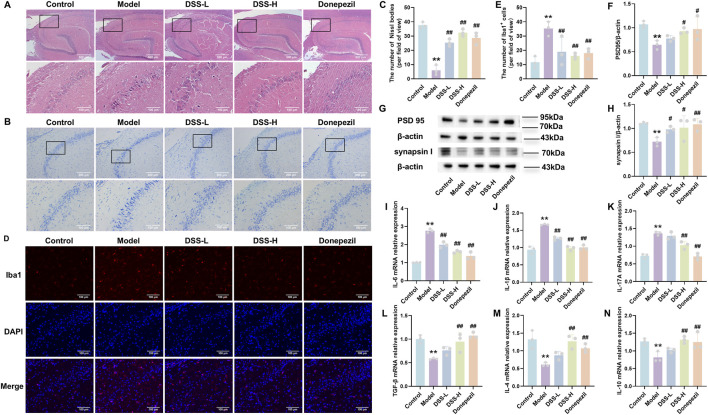
DSS ameliorates neuronal damage and alleviates neuroinflammation in AD rats. **(A)** Representative H&E staining images of hippocampal tissue and the CA2 region (×40 and ×200 magnification). **(B)** Representative Nissl staining images of hippocampal tissue and the CA2 region (×100 and ×200 magnification). **(C)** Statistical analysis of Nissl body count per field of view. **(D)** Immunofluorescence co-localization images of Iba1 (red) and DAPI (blue) in the hippocampal CA2 region. **(E)** Quantification of Iba1^+^ activated microglia across groups per field of view. **(F)** Quantitative analysis of PSD 95 protein expression levels. **(G)** Representative Western blot bands of PSD 95, synaptophysin I proteins in hippocampal tissue. **(H)** Quantitative analysis of synaptophysin I protein expression levels. **(I–K)** mRNA expression levels of the pro-inflammatory cytokines IL-6, IL-1β, and IL-17A in brain tissue. **(L–N)** mRNA expression levels of the anti-inflammatory cytokines TGF-β, IL-4, and IL-10 in brain tissue. Data are presented as mean ± SD (n = 3). *p < 0.05, **p < 0.01 vs. control group; #*p* < 0.05, ##*p* < 0.01 vs. model group.

To systematically assess the neuropathological impact of DSS in D-galactose-induced AD rats, histological analyses of the hippocampal CA2 and CA3 regions were performed. Hematoxylin and eosin (H&E) staining (Figure 2A) showed well-organized neurons with clear nuclear morphology in controls, whereas the model group exhibited disordered neuronal arrangement with signs of injury such as pyknosis and nuclear condensation. Treatment with DSS (both doses) and donepezil restored neuronal order and reduced nuclear condensation and neuronal loss. Nissl staining (Figure 2B) revealed reduced neuronal count, irregular arrangement, and diminished Nissl body content in the model group; DSS intervention counteracted these changes, increasing neuronal density and Nissl body restoration (*p* < 0.01, Figure 2C), indicating effective mitigation of D-galactose-induced structural neuronal damage. To explore DSS’s regulatory effect on neuroinflammation, we evaluated microglial activation and inflammatory cytokine expression. Immunofluorescence staining (Figure 2D) showed a significant increase of Iba1^+^ activated microglia in the model group (*p* < 0.01), which was significantly suppressed by DSS and donepezil treatments (*p* < 0.01, Figure 2E). Western blot analysis was performed to detect the expression of AD-related pathological markers synaptic proteins PSD95 and Synapsin I in the hippocampus of each group. Compared with the control group, the model group exhibited significantly decreased expression levels of both PSD95 (*p* < 0.01) and Synapsin I (*p* < 0.01). Conversely, treatment with a high dose of DSS restored the expression of these proteins compared to the model group (*p* < 0.05) (Figures 2F–H). qPCR analysis revealed elevated mRNA levels of pro-inflammatory cytokines IL-6, IL-1β, and IL-17A and decreased expression of anti-inflammatory cytokines TGF-β, IL-4, and IL-10 in the hippocampus of model rats. DSS treatment significantly reversed these expression patterns (*p* < 0.01, Figures 2I–N), with the high-dose DSS group demonstrating more pronounced effects.

### 3.3 Network pharmacology analysis of DSS in alleviating AD-Related neuroinflammation

**FIGURE 3:**
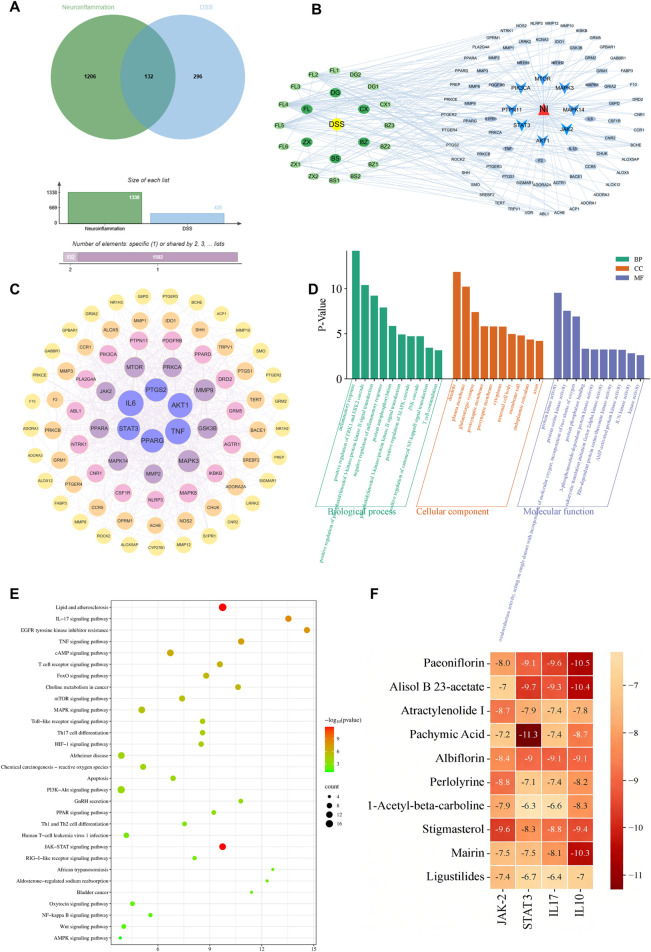
Network pharmacological analysis of DSS against neuroinflammation. **(A)** Venn diagram illustrating the overlap between potential targets of DSS and neuroinflammation-related genes. **(B)** Compound-target-disease interaction network. **(C)** Protein-protein interaction (PPI) network of the core overlapping targets. **(D,E)** Functional enrichment analysis of the core targets: **(D)** Gene Ontology (GO) terms and **(E)** Kyoto Encyclopedia of Genes and Genomes (KEGG) pathways. **(F)** Heatmap of binding affinity between key active compounds and core targets obtained from molecular docking.

To elucidate the potential mechanisms by which DSS counteracts neuroinflammation, network pharmacology analysis was conducted. Thirty-two active compounds were identified from DSS (Supplementary Table S3), corresponding to 296 potential drug targets, while 1206 neuroinflammation-related targets were retrieved from disease databases. Venn diagram analysis (Venny 2.1) revealed 132 overlapping targets (Figure 3A; Supplementary Table S4), highlighting potential DSS intervention sites. An “Active Component-Target-Disease” network was constructed (Figure 3B; Supplementary Tables S5, S6), demonstrating the characteristic multi-component, multi-target synergy of DSS. Protein-protein interaction (PPI) network analysis identified core hub targets including AKT1, TNF-α, STAT3, and JAK2 (Figure 3C; Supplementary Tables S7, S8), suggesting pivotal roles in DSS-mediated neuroinflammation regulation. GO enrichment analysis showed significant involvement in biological processes (BP) such as inflammatory response, JNK cascade activation, and T cell costimulation; cellular components (CC) related primarily to dendrites and plasma membranes; and molecular functions (MF) centered on protein kinase activity (Figure 3D; Supplementary Table S9). KEGG pathway analysis indicated significant enrichment in immune-related pathways, including JAK-STAT, IL-17, T cell receptor signaling, and differentiation of Th1/Th2 and Th17 cells (Figure 3E; Supplementary Table S10). Molecular docking revealed that ten key DSS compounds exhibited strong binding affinities (binding energy < −5.0 kcal/mol) toward JAK2, STAT3, IL17, and IL10 (Figure 3F; Supplementary Figure S1), indicating that multiple active constituents of DSS may target these kinases. Collectively, these data suggest that DSS ameliorates AD-associated neuroinflammation primarily via modulation of T cell immunity and related signaling pathways.

### 3.4 Serum untargeted metabolomics reveals DSS modulates key metabolic pathways affecting the JAK2/STAT3 axis

**FIGURE 4:**
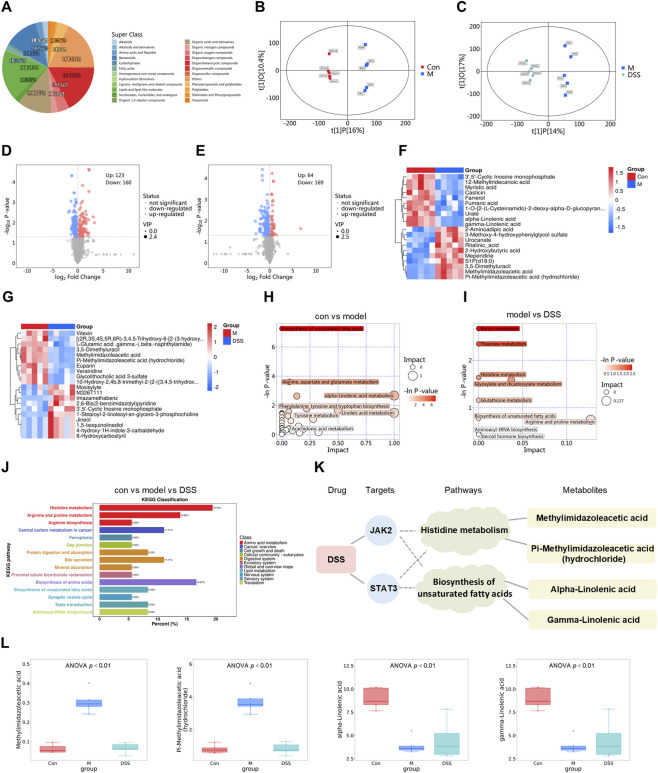
DSS ameliorates metabolic disturbances in rats with neuroinflammation. **(A)** Pie chart showing the classification and distribution of identified metabolites. **(B,C)** Orthogonal partial least squares-discriminant analysis (OPLS-DA) score plots for **(B)** Control vs. Model and **(C)** Model vs. DSS groups. **(D,E)** Volcano plots of differentially abundant metabolites for **(D)** Control vs. Model and **(E)** Model vs. DSS comparisons. Metabolites farther from the origin on the x-axis represent greater fold-change. Colored points denote significantly upregulated (red) or downregulated (blue) metabolites based on threshold criteria; gray points indicate non-significant changes. **(F)** Hierarchical clustering heatmap of differentially abundant metabolites between the Control and Model groups. **(G)** Hierarchical clustering heatmap of differentially abundant metabolites between the Model and DSS groups. **(H)** Bubble plot of KEGG pathways enriched with differentially abundant metabolites between the Control and Model groups. **(I)** Bubble plot of KEGG pathways enriched with differentially abundant metabolites between the Model and DSS groups. **(J)** Bar chart of KEGG pathway categories for the differentially abundant metabolites. **(K)** Connections between key metabolites and targets. **(L)** Statistical plots of the abundance of the 4 key metabolites.

To further explore the metabolic mechanisms underlying DSS-mediated cognitive improvement, untargeted metabolomic profiling of rat serum was performed using UHPLC-QE-MS. Quality control confirmed data reliability. Thirteen metabolite categories were identified, with lipids and lipid-like molecules comprising the largest proportion (17.916%) (Figure 4A; Supplementary Table S11). Orthogonal partial least squares-discriminant analysis (OPLS-DA) revealed clear separations among Control, Model, and DSS groups, indicating significant metabolic disturbances in AD rats that were partly reversed by DSS (Figures 4B,C). Volcano plot analysis identified 123 upregulated and 160 downregulated metabolites in the Model vs. Control comparison, while DSS treatment led to 64 upregulated and 169 downregulated metabolites relative to the Model group (Figures 4D,E). Hierarchical clustering confirmed that DSS reversed dysregulation of key metabolites, including polyunsaturated fatty acids (docosahexaenoic acid, α-linolenic acid, γ-linolenic acid) and the second messenger cAMP, implying a multi-pathway coordination role (Figures 4F,G; Supplementary Tables S12, S13) KEGG pathway enrichment showed significant involvement of fatty acid and amino acid metabolism pathways—particularly alanine, aspartate and glutamate metabolism, and nicotinate and nicotinamide metabolism (Figures 4H–J; Supplementary Tables S14–S16). Cross-validation of network pharmacology outputs and metabolomics datasets revealed that core targets JAK2 and STAT3 were linked to four key metabolites (methylimidazoleacetic acid, pi-methylimidazoleacetic acid (hydrochloride), α-linolenic acid, γ-linolenic acid), which participate in histidine metabolism, unsaturated fatty acid biosynthesis, and fatty acid metabolism (Figures 4K,L; Supplementary Table S4). Together with network pharmacology’s designation of JAK2 as a hub target, these observations imply that DSS could regulate the JAK2/STAT3 signaling axis through targeted modulation of these metabolic cascades.

### 3.5 DSS regulates peripheral Th17/Treg -related immune dysregulation

**FIGURE 5:**
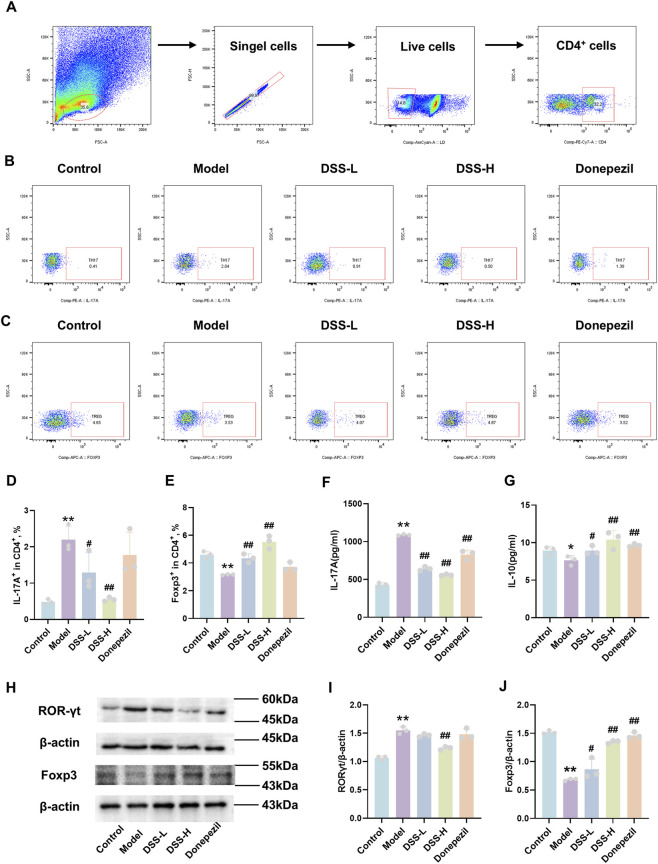
DSS modulates the peripheral and intracerebral Th17/Treg -related immune dysregulation. **(A)** Gating strategy for Th17 and Treg populations in splenic CD4^+^T cells by flow cytometry. **(B,D)** Quantitative analysis of the percentage of Th17 cells (CD4^+^IL-17A^+^) in the spleen. **(C,E)** Quantitative analysis of the percentage of Treg cells (CD4^+^Foxp3^+^) in the spleen. **(F)** Serum concentration of IL-17A. **(G)** Serum concentration of IL-10. **(H)** Representative Western blot bands of RORγt and Foxp3 proteins in hippocampal tissue. **(I)** Semi-quantitative analysis of RORγt protein expression. **(J)** Semi-quantitative analysis of Foxp3 protein expression. Data are presented as mean ± SD (n = 3). *p < 0.05, **p < 0.01 vs. control group; #*p* < 0.05, ##*p* < 0.01 vs. model group.

To determine whether DSS influences the Th17/Treg balance in AD rats, flow cytometric analysis of splenic CD4^+^ T cell subsets was conducted. Compared with controls, model rats showed a significant increase in Th17 cells (CD4^+^IL-17A^+^) and a marked decrease in Treg cells (CD4^+^Foxp3^+^) (*p* < 0.01). DSS treatment significantly ameliorated this imbalance (*p* < 0.05, Figures 5A–E). ELISA further demonstrated that DSS lowered serum IL-17A levels (*p* < 0.01) while elevating IL-10 concentrations (*p* < 0.05) in model rats (Figures 5F,G). These results indicate that DSS effectively restores Th17/Treg cell ratios and associated cytokine profiles in both peripheral immune organs and systemic circulation.

### 3.6 DSS restores central Th17/Treg -related immune dysregulation

**FIGURE 6:**
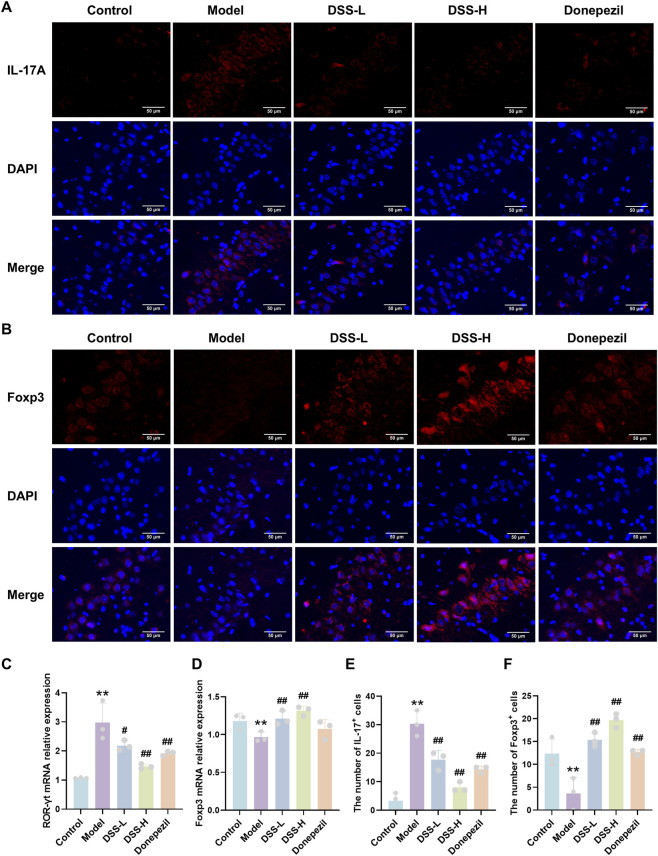
DSS regulates the expression and distribution of intracerebral Th17/Treg-related molecules. **(A)** Representative immunofluorescence staining images of IL-17A in the hippocampal CA2 region. **(B)** Representative immunofluorescence staining images of Foxp3 in the hippocampal CA2 region. **(C)** Relative mRNA expression level of RORγt. **(D)** Relative mRNA expression level of Foxp3. **(E)** Quantification of IL-17A-positive cells. **(F)** Quantification of Foxp3-positive cells. Data are presented as mean ± SD (n = 3). *p < 0.05, **p < 0.01 vs. control group; #*p* < 0.05, ##*p* < 0.01 vs. model group.

The regulatory effect of DSS on central nervous system Th17/Treg -related immune dysregulation was assessed via qRT-PCR and Western blot of hippocampal tissue. The model group exhibited significantly upregulated mRNA and protein levels of RORγt (*p* < 0.01), the Th17-specific transcription factor, accompanied by downregulation of Foxp3 (*p* < 0.01), the key Treg transcription factor. DSS-H treatment reversed these changes, significantly decreasing RORγt and increasing Foxp3 expression (*p* < 0.01) (Figures 5H–J, (Figures 6C,D). Immunofluorescence staining further confirmed increased IL-17A and decreased Foxp3 signals in the hippocampal CA2 region of the model group (*p* < 0.01), both of which were significantly normalized by DSS (Figures 6A,B,E,F). These findings demonstrate that DSS not only corrects Th17/Treg -related immune dysregulation in peripheral immune organs and circulation but also directly modulates key immune molecules in the brain, thereby restoring systemic and central immune dysregulation and alleviating AD-associated neuroinflammation.

### 3.7 DSS inhibits JAK2/STAT3 pathway activation

**FIGURE 7:**
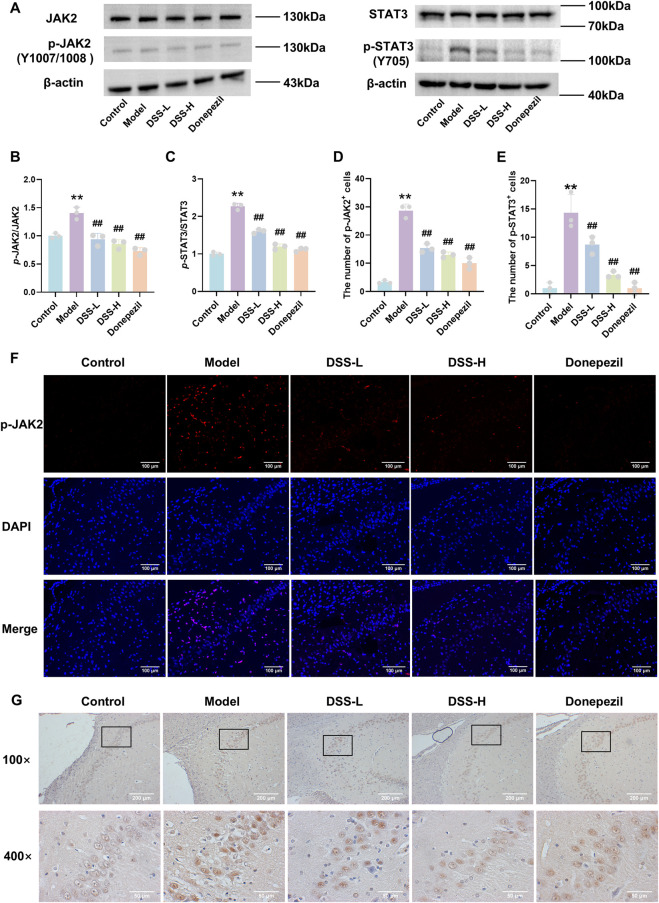
DSS alleviates neuroinflammation in AD rats via inhibition of the JAK2/STAT3 signaling pathway. **(A)** Representative Western blot bands of JAK2, p-JAK2, STAT3, and p-STAT3 proteins in hippocampal tissue. **(B)** Quantitative analysis of p-JAK2 protein expression levels. **(C)** Quantitative analysis of p-STAT3 protein expression levels. **(D)** Statistical analysis of p-JAK2-positive cells by immunofluorescence (IF). **(E)** Statistical analysis of p-STAT3-positive cells by immunohistochemistry (IHC). **(F)** Representative immunofluorescence images showing p-JAK2 (red) and DAPI (blue) in the hippocampal CA2 region. **(G)** Representative immunohistochemistry (IHC) images of p-STAT3 expression in the hippocampus and the CA2 region (×100 and ×400 magnification). Data are presented as mean ± SD (n = 3). *p < 0.05, **p < 0.01 vs. control group; #*p* < 0.05, ##*p* < 0.01 vs. model group.

Given the implication of the JAK/STAT pathway in DSS’s anti-neuroinflammatory effects from network pharmacology and metabolomics analyses, we experimentally validated this mechanism. Western blot data showed significantly elevated phosphorylated JAK2 (p-JAK2) and STAT3 (p-STAT3) protein levels in hippocampal tissue of model rats compared to controls (*p* < 0.01), while total JAK2 and STAT3 levels remained unchanged. Treatment with DSS markedly reduced p-JAK2 and p-STAT3 levels (*p* < 0.01), indicating selective inhibition of phosphorylation-dependent activation of the pathway (Figures 7A–C). Immunofluorescence and immunohistochemistry analyses corroborated these findings, with increased p-JAK2 and p-STAT3 signals observed in the model group hippocampus, which were significantly diminished following DSS treatment (*p* < 0.01, Figures 7D–G). Collectively, these results indicate that DSS alleviates neuroinflammation in AD rats may via suppression of the JAK2/STAT3 signaling pathway.

## 4 Discussion

AD is a multifactorial neurodegenerative disorder with complex pathology and limited effective treatments (Scheltens et al., 2021). Neuroinflammation, involving interplay between central and peripheral immune systems, is a key driver. The Th17/Treg balance is critical (Wang et al., 2024), as Th17 cells promote inflammation while Treg cells maintain homeostasis (Yang et al., 2020). In recent years, TCM has demonstrated unique advantages in improving cognitive function and preventing or treating AD. Its holistic regulatory characteristics—multi-component, multi-target, and multi-pathway—along with relatively low toxicity and side effects, have attracted considerable attention (Ding et al., 2022). DSS, a classic TCM formula, has been confirmed by multiple studies to have the potential to improve cognitive function and AD-related pathologies (Kim and Cho, 2020). Based on a D-galactose-induced AD rat model, which simulates core features of AD such as enhanced brain inflammation, declined immune function, and cognitive impairment (Daroi et al., 2022), we demonstrated through MWM and open field tests, combined with hippocampal histopathological and AD-related pathological markers (PSD 95, synaptophysin I) analysis, that DSS significantly improved learning and memory abilities and alleviated neuronal structural damage in AD model rats. DSS intervention markedly suppressed the overactivation of microglia in the brain, reduced the levels of pro-inflammatory factors IL-6, IL-1β, and IL-17A, indicating its ability to modulate neuroinflammation. Furthermore, we observed disturbances in the Th17/Treg -related immune dysregulation both peripherally and centrally. Specifically, in the model group, Th17 cells and related factors were elevated in the spleen and serum, while Treg cells and associated anti-inflammatory factors were decreased; in brain tissues, RORγt expression was increased whereas Foxp3 expression was decreased. DSS treatment effectively reversed Th17/Treg -related immune dysregulation, consistent with findings from other studies (Kaushal et al., 2024; Kawata et al., 2021).

Under physiological conditions, the blood-brain barrier (BBB) effectively prevents peripheral lymphocytes from entering the brain parenchyma (Castellani et al., 2023). However, structural and functional impairment of the BBB represents one of the critical pathological features in the early stages of AD and is closely associated with its pathogenesis (Szablewski, 2021; Bettcher et al., 2021; Cisbani and Rivest, 2021). Damage to the BBB leads to increased permeability and loosened inter-endothelial junctions, allowing T lymphocytes to infiltrate the central nervous system through defective sites, particularly accumulating in AD-related brain regions such as the hippocampus, thereby promoting further recruitment and activation of inflammatory cells (Berron et al., 2021). Simultaneously, chemokines and pro-inflammatory cytokines released by local inflammatory cells after BBB disruption can further attract and activate T cells, forming a positive-feedback loop that facilitates T-cell migration and infiltration (Cheng et al., 2021; Mietelska-Porowska and Wojda, 2017).Neuroinflammation in AD is characterized by sustained microglial activation and excessive production of inflammatory factors, triggering a complex immunoinflammatory cascade in which the balance between Th17 and Treg cells plays a crucial regulatory role (Cao et al., 2022). Upregulation of inflammatory factors promotes the expression of ROR-γt, which in turn induces the Th17 phenotype and IL-17 production (Tsiogkas et al., 2022). In contrast, Treg cells suppress immune responses by expressing the transcription factor Foxp3 and secreting IL-10 and TGF-β (Zhang et al., 2020). The present study demonstrated that the expression of the Th17-specific transcription factor RORγt and its key effector cytokine IL-17A were significantly increased in the hippocampal tissues of AD model rats, suggesting that peripherally derived Th17 cells may infiltrate the brain through the impaired BBB. Concurrently, elevated levels of IL-17A in the spleen and serum further indicated systemic immune dysregulation in AD. Conversely, the expression of the Treg-associated transcription factor Foxp3 and the anti-inflammatory cytokine IL-10 was markedly reduced, confirming that neuroinflammation in AD is accompanied by a significant disruption of Th17/Treg immune homeostasis. Intervention with DSS effectively reversed these abnormal changes and rescue Th17/Treg -associated immune dysregulation. This suggests that DSS may alleviate AD neuroinflammatory progression at multiple levels through coordinated modulation of peripheral and central immune responses.

Based on network pharmacology analysis, this study identified 32 potential active components in DSS, with key protein targets including AKT1, STAT3, and TNF. Further analysis indicated that these targets are significantly enriched in inflammation-related signaling pathways such as JAK-STAT and IL-17. PPI network analysis suggested that DSS possesses notable inhibitory potential against JAK2 and STAT3. Additionally, metabolomic analysis revealed that DSS effectively reversed abnormal changes in multiple metabolites in the brains of AD model rats. KEGG pathway enrichment analysis demonstrated that the differential metabolites regulated by DSS are primarily involved in core biological processes such as fatty acid metabolism and amino acid metabolism, which are closely related to the regulatory mechanisms of neuroinflammation (Zhou et al., 2025; Kumar Dash et al., 2025). Notably, alanine, aspartate, and glutamate metabolism as well as nicotinate and nicotinamide metabolism were identified as key metabolic nodes through which DSS exerts its effects. These metabolic pathways exhibit close interactions with the JAK/STAT signaling pathway. Specifically, glutamate metabolites can influence the functional behavior of immune cells by modulating their energy metabolic state (Shen and Zhang, 2025). Meanwhile, nicotinate metabolism regulates intracellular NAD^+^ levels and the activity of the Sirtuin protein family, thereby indirectly affecting the activation status of the JAK/STAT signaling pathway and subsequently modulating inflammatory responses and immune cell differentiation processes (Daver and Cortes, 2012). Therefore, the therapeutic efficacy of DSS against AD-associated neuroinflammation may stem from its multi-component, multi-target synergistic characteristics, particularly its ability to intervene in the JAK/STAT signaling pathway, highlighting the unique advantage of TCM formulations in exerting therapeutic effects through integrated multi-pathway regulation.

The JAK/STAT signaling pathway plays a pivotal regulatory role in the neuroinflammatory process of neurodegenerative diseases such as AD (Jain et al., 2021). This pathway participates in the pathological progression of neuroinflammation by initiating innate immune responses and coordinating adaptive immune mechanisms. Recent studies have shown that inhibiting the phosphorylation level of the JAK2/STAT3 pathway can effectively alleviate neuroinflammatory responses (Weng et al., 2017). From a molecular mechanistic perspective, activation of the JAK2/STAT3 signaling pathway is initiated by the binding of cytokines (e.g., IL-6) to their receptors (e.g., the IL-6R/gp130 complex). This binding event induces autophosphorylation of JAK2, which is associated with the intracellular domain of the receptor, subsequently phosphorylating specific tyrosine residues on the receptor to create docking sites for STAT3. Once STAT3 binds to these phosphorylated sites, its Tyr705 residue is phosphorylated by JAK2, leading to STAT3 dimerization and translocation into the nucleus. Within the nucleus, the STAT3 dimer binds to specific DNA response elements, regulating the transcription of numerous genes involved in processes such as cell survival, proliferation, and inflammatory responses (O'Shea et al., 2015). Notably, the JAK2/STAT3 signaling pathway plays a central regulatory role in maintaining Th17/Treg immune balance (Yang et al., 2022). Activation of this pathway can drive Th17 cell differentiation and the secretion of the pro-inflammatory cytokine IL-17. Following nuclear translocation, STAT3 upregulates the expression of RORγt, a key transcription factor for Th17 cells, thereby promoting Th17 cell proliferation and amplifying pro-inflammatory responses (Zhao et al., 2021). In the neuroinflammatory milieu of AD, key cytokines such as IL-6 persistently activate the JAK2/STAT3 pathway, simultaneously promoting Th17 cell differentiation and suppressing Treg cell function, ultimately leading to Th17/Treg imbalance (Alanazi et al., 2023; Mao et al., 2019; Liu et al., 2024). Therefore, aberrant activation of the JAK/STAT signaling pathway is an important factor in disrupting immune homeostasis and exacerbating AD neuroinflammation. This study found that in D-galactose-induced AD rat models, the JAK2/STAT3 inflammatory pathway was significantly activated, as evidenced by markedly elevated JAK2 phosphorylation levels accompanied by a pronounced increase in inflammatory cytokine levels. Following DSS intervention, not only were inflammatory cytokine levels significantly reduced, but the activation of the JAK2/STAT3 pathway was also notably inhibited, suggesting that DSS may exert its anti-neuroinflammatory effects by suppressing the JAK2/STAT3 pathway.

Despite the strengths of this study, several limitations should be acknowledged. First, the D-galactose-induced model primarily reflects aging-related cognitive impairment accompanied by oxidative stress and neuroinflammation, rather than fully recapitulating the classical pathological hallmarks of AD, such as Aβ deposition and tau hyperphosphorylation. Therefore, caution is warranted when extrapolating these findings to human AD pathology. Second, behavioral experiments were performed with a modest sample size (n = 5 per group), which may limit statistical power for detecting small effects; larger cohorts will be valuable for confirming these findings. In addition, although alterations in Th17- and Treg-associated immune dysregulation were observed in both the central nervous system and peripheral circulation, the present study did not directly trace the cellular origin of these immune cells or conclusively demonstrate their migration across the blood–brain barrier. Future studies incorporating lineage tracing or adoptive transfer approaches may help clarify the dynamic interactions between peripheral immune cells and the central nervous system. Finally, while our data support an association between JAK2/STAT3 pathway inhibition and immunomodulatory effects of DSS, causal relationships require further validation using genetic or pharmacological manipulation of this signaling axis.

## 5 Conclusion

**FIGURE 8:**
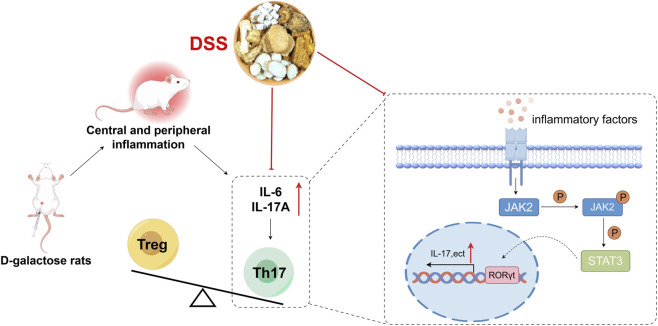
DSS rescue Th17/Treg -related immune dysregulation both in the brain and periphery in a D-galactose-induced Alzheimer’s disease rat model. DSS exerts its anti-neuroinflammatory effects by inhibiting JAK2 and STAT3 phosphorylation, reducing their nuclear translocation, and consequently suppressing Th17 differentiation and pro-inflammatory cytokine production.

This study demonstrates that DSS is associated with improved cognitive performance and attenuated neuroinflammatory responses in a D-galactose-induced AD rat model. Our findings suggest that the neuroprotective effects of DSS are linked to multi-target and multi-pathway regulatory actions, including modulation of JAK2/STAT3-associated inflammatory signaling, alterations in peripheral and central Th17- and Treg-related immune responses, and changes in metabolic profiles. (Figure 8). Although causal relationships cannot be definitively established in the present study, these results support the potential relevance of DSS for alleviating neuroinflammation-associated cognitive dysfunction and highlight the integrated regulatory characteristics of traditional Chinese medicine formulations.

## Data Availability

The original contributions presented in the study are included in the article/Supplementary Material, further inquiries can be directed to the corresponding authors.

## Glossary

Aβ: β-amyloid
AD: Alzheimer’s disease
AKT1: Protein Kinase B1
ANOVA: analysis of variance
BBB: blood-brain barrier
BCA: Bicinchoninic Acid
BE: binding energies
BP: biological processes
BSA: bovine serum albumin
CA2: Cornu Ammonis Area 2
CA3: Cornu Ammonis Area 3
cAMP: 3′,5′-cyclic adenosine monophosphate
CC: cellular components
DAB: 3,3′-Diaminobenzidine
DAVID: Database for Annotation Visualization and Integrated Discovery
DAPI: 4′,6-diamidino-2-phenylindole
DHA: docosahexaenoic acid
DL: drug-likeness
DSS: Danggui Shaoyao San
ELISA: enzyme-linked immunosorbent assay
FC: fold change
FDA: Food and Drug Administration
Foxp3: forkhead box protein 3
GO: Gene Ontology
HE: hematoxylin and eosin
HMDB: Human Metabolome Database
Iba1: ionized calcium binding adaptor molecule 1
IL-1β: interleukin-1 beta
IL-17A: Interleukin-17A
IL-10: interleukin-10
IL-6: interleukin-6
JAK/STAT: Janus kinase/Signal Transducer and Activator of Transcription
JAK2: janus kinase 2
JNK: c-Jun N-terminal Kinase
KEGG: Kyoto Encyclopedia of Genes and Genomes
LC-MS: Liquid Chromatography-Mass Spectrometry
MF: molecular functions
MWM: Morris water maze
NAD: Nicotinamide Adenine Dinucleotide
NCBI: National Center for Biotechnology Information
NF-κB: Nuclear Factor-kappa B
NLRP3: NOD-like Receptor Pyrin Domain-Containing Protein 3
NO: nitric oxide
OB: oral bioavailability
OFT: open field test
OMIM: Online Mendelian Inheritance in Man
OPLS-DA: orthogonal partial least squares discriminant analysis
PBS: phosphate-buffered saline
PCA: principal component analysis
p-JAK2: Phosphorylated Janus Kinase 2
PPI: protein–protein interaction
PSD95: postsynaptic density protein 95
p-STAT3: Phosphorylated Signal Transducer and Activator of Transcription 3
PubChem: PubChem Database
PVDF: polyvinylidene fluoride
qPCR: quantitative polymerase chain reaction
RCSB PDB: Research Collaboratory for Structural Bioinformatics Protein Data Bank
RIPA: Radio-Immunoprecipitation Assay Buffer
RORγt: retinoid-related orphan receptor gamma t
SDS-PAGE: Sodium Dodecyl Sulfate-Polyacrylamide Gel Electrophoresis
STAT3: signal transducer and activator of transcription 3
STRING: Search Tool for the Retrieval of Interacting Genes/Proteins Database
SYBR: SYBR Green
TCM: Traditional Chinese Medicine
TCMSP: Traditional Chinese Medicine Systems Pharmacology Database and Analysis Platform
Th1: T helper cell 1
Th1: T helper cell 2
Th17: T helper cell 17
Treg: Regulatory T cell
TNF-α: tumor necrosis factor alpha
TGF-β: Transforming Growth Factor-beta
UHPLC-QE-MS: Ultra-High Performance Liquid Chromatography-Quadrupole Electrostatic Field Orbitrap Mass Spectrometry
UPLC: ultra-performance liquid chromatography
USA: United States of America
VIP: variable importance in projection
