# Supplementary material for: Danggui Shaoyao San ameliorates neuroinflammation in a D-galactose-induced Alzheimer’s disease rat model by suppressing the JAK2/STAT3 pathway and modulating Th17/Treg -related immune dysregulation
Source: Front Cell Dev Biol. 2026 Feb 25;14:1763180. doi: 10.3389/fcell.2026.1763180 (PMC12975871; doi:10.3389/fcell.2026.1763180)
Supplement: Supplementary file 1 [file Supplementaryfile1.docx]

Supplementary Material

# Supplementary Tables

## Supplementary Table S1. Chemicals and antibody for flow cytometry.

| **Name** | **Catalog#** | **Source** |
| --- | --- | --- |
| Lysing Buffer | 555899 | BD Pharmingen |
| Leukocyte Activation Cocktail | 550583 | BD GolgiPlug |
| Fixable Viability Stain 510 | 544404 | BD Pharmingen |
| Purified Mouse Anti-Rat CD32(D34-485) | 550250 | BD Pharmingen |
| PE-Cy5 Mouse Anti-Rat CD4(OX-35) | 541558 | BD Pharmingen |
| Transcription Factor Buffer Set | 542554 | BD Pharmingen |
| ANTI-MO/RT FOXP3 FJK-14S APC | 15-5553-82 | Thermo |
| ANTI-MO/RT IL-15A EBIO15B5 PE | 12-5155-81 | Thermo |

## Supplementary Table S2. The list of Network pharmacology Website.

| **Name** | **Website** |
| --- | --- |
| TCMSP | https://old.tcmsp-e.com/tcmsp.php |
| PubChem | https://pubchem.ncbi.nlm.nih.gov/ |
| SwissTargetPrediction | http://www.swisstargetprediction.ch/ |
| Venny | http://www.bioinformatics.com.cn/static/others/jvenn/example.html |
| GeneCards | https://www.genecards.org/ |
| OMIM | https://www.omim.org/ |
| STRING | (http://string-db.org/) |
| Drunbank | https://go.drugbank.com/ |
| STRING | https://cn.string-db.org/ |
| DAVID | https://david.ncifcrf.gov |

## Supplementary Table S3. The list of Chemical components of DSS.

| **NO** | **Compound** | **OB％** | **BBB** | **DL** | **Chinese herb** |
| --- | --- | --- | --- | --- | --- |
| M1 | (-)-alpha-cedrene | 55.54 | 0.1 | 2.14 | Baishao |
| M2 | Methyl linolelaidate | 41.93 | 0.15 | 1.9 | Baishao |
| M3 | DBP | 44.54 | 0.54 | 0.13 | Baishao |
| M4 | [sitosterol](https://www.tcmsp-e.com/molecule.php?qn=359) | 34.91 | 0.85 | 0.55 | Baishao |
| M5 | Mairin | 55.38 | 0.22 | 0.58 | Baishao |
| M4 | [a-Longipinene](https://www.tcmsp-e.com/molecule.php?qn=25) | 53.24 | 2.14 | 0.12 | Baizhu |
| M5 | [a-Amyrin](https://www.tcmsp-e.com/molecule.php?qn=28) | 39.51 | 1.28 | 0.54 | Baizhu |
| M8 | [3β-acetoxyatractylone](https://www.tcmsp-e.com/molecule.php?qn=49) | 54.05 | 1.08 | 0.22 | Baizhu |
| M9 | 8β-ethoxy atractylenolide Ⅲ | 35.95 | 1.12 | 0.21 | Baizhu |
| M10 | [atractylenolide i](https://www.tcmsp-e.com/molecule.php?qn=43) | 35.35 | 1.29 | 0.15 | Baizhu |
| M9 | [atractylenolideII](https://www.tcmsp-e.com/molecule.php?qn=44) | 45.5 | 1.35 | 0.15 | Baizhu |
| M12 | atractylenolide iii | 48.9 | 0.43 | 0.15 | Baizhu |
| M13 | [DIBP](https://www.tcmsp-e.com/molecule.php?qn=57) | 49.43 | 0.48 | 0.13 | Baizhu |
| M14 | [Akridin](https://www.tcmsp-e.com/molecule.php?qn=38) | 33.51 | 1.53 | 0.1 | Baizhu |
| M15 | [EIC](https://www.tcmsp-e.com/molecule.php?qn=131) | 41.9 | 0.9 | 0.14 | Chuanxiong |
| M14 | 49050_FLUKA | 85.51 | 1.44 | 0.12 | Chuanxiong |
| M15 | Mandenol | 42 | 1.14 | 0.19 | Chuanxiong |
| M18 | METHYL LINOLEATE | 41.93 | 1.08 | 0.15 | Chuanxiong |
| M19 | (+)-ALPHA-FUNEBRENE | 52.85 | 2.08 | 0.1 | Chuanxiong |
| M20 | betea-CUBEBENE | 32.14 | 2.13 | 0.9 | Chuanxiong |
| M21 | Perlolyrine | 45.95 | 0.15 | 0.25 | Chuanxiong |
| M22 | 1-Acetyl-beta-carboline | 45.12 | 0.81 | 0.13 | Chuanxiong |
| M23 | Aromadendrene oxide 2 | 45.1 | 1.82 | 0.14 | Chuanxiong |
| M24 | Cedrene | 51.14 | 2.12 | 0.9 | Chuanxiong |
| M25 | beta-sitosterol | 34.91 | 1.32 | 0.99 | Danggui |
| M24 | Stigmasterol | 43.83 | 1 | 0.54 | Danggui |
| M25 | [2,4-di(phenyl)thiopyran-4-thione](https://www.tcmsp-e.com/molecule.php?qn=8259) | 49.13 | 1.29 | 0.15 | Danggui |
| M28 | cis-Thujopsene | 54.43 | 2.24 | 0.12 | Danggui |
| M29 | ergosta-5,22E-dien-3beta-ol | 43.51 | 0.91 | 0.52 | Fuling |
| M30 | [hederagenin](https://www.tcmsp-e.com/molecule.php?qn=296) | 34.91 | 0.94 | 0.55 | Fuling |
| M31 | sitosterol | 34.91 | 0.85 | 0.55 | Zexie |
| M32 | [(1S,3aR,4R,8aS)-5-isopropyl-1,4-dimethyl-2,3,3a,5,4,8a-hexahydroazulene-1,4-diol](https://www.tcmsp-e.com/molecule.php?qn=827) | 59.48 | 0.32 | 0.1 | Zexie |

## Supplementary Table S4. Intersection targets of DSS and Neuroinflammation.

## Supplementary Table S5. Protein‒protein interaction (PPI) network cluster module.

## Supplementary Table S6. Core targets from the Protein-Protein Interaction (PPI) network ranked by degree centrality.

## Supplementary Table S7. Interaction Data for the Compound-Target-Disease Network.

## Supplementary Table S8. Definition of Node Types in the Compound-Target-Disease Network.

## Supplementary Table S9. Gene Ontology (GO) enrichment analysis of the predicted targets.

## Supplementary Table S10. KEGG enrichment analysis of the predicted targets.

## Supplementary Table S11. The pie data of total metabolites in serum.

## Supplementary Table S12. The differential metabolites in serum between group control and group model. *p<0.05, **p<0.01.

## Supplementary Table S13. The differential metabolites in serum between group model and group DSS. *p<0.05, **p<0.01.

## Supplementary Table S14. The enriched metabolic pathways of differential metabolites in serum between group control and group model.

## Supplementary Table S15. The enriched metabolic pathways of differential metabolites in serum between group model and group DSS.

## Supplementary Table S16. The enriched metabolic pathways of differential metabolites in serum among group control and group model and group DSS.

## **Supplementary Table S17.** Statistical plots of the abundance of the 4 key metabolites.

# Supplementary Figure

**
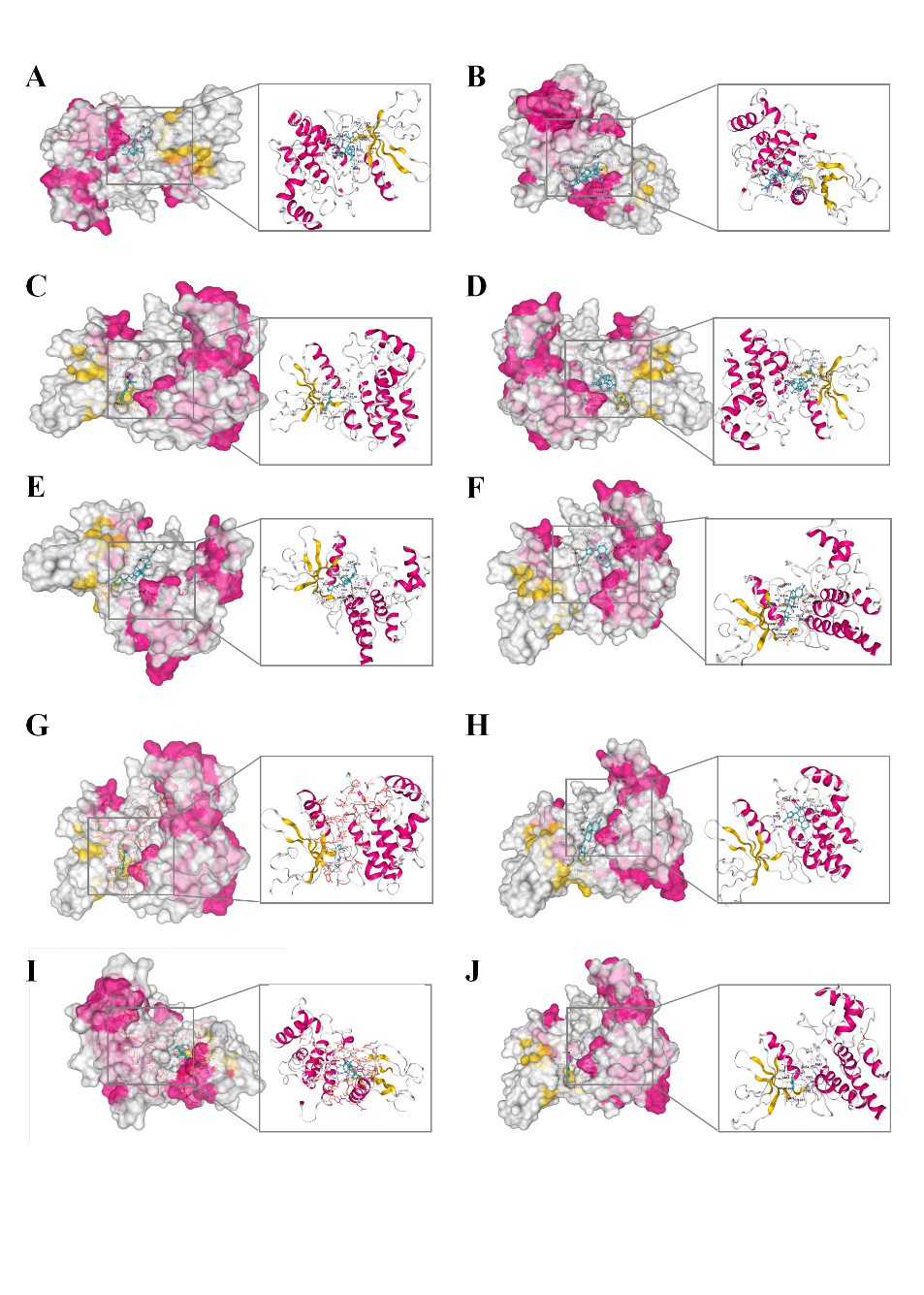
**

**Supplementary Figure S1.** The main components of Danggui Shaoyao San and the JAK2 docking complex. (A) Paeoniflorin-JAK2 (B) Alisol B 23-acetate-JAK2 (C) Atractylenolide I-JAK2 (D) Pachymic Acid-JAK2 (E) Albiflorin-JAK2 (F) Perlolyrine-JAK2 (G) 1-Acetyl-beta-carboline-JAK2 (H) Stigmasterol-JAK2 (I) Mairin-JAK2 (J) Ligustilides-JAK2
